# Determinants of Structural Joint Damage in Psoriatic Arthritis: Limited Association with Disease Activity and Modest Link with Health Impact

**DOI:** 10.3390/jcm15041506

**Published:** 2026-02-14

**Authors:** Paula Alvarez, Stefanie Burger, Estefanía Pardo, Ignacio Braña, Marta Loredo, Norma Callejas, Sara Alonso, Mercedes Alperi, Rubén Queiro

**Affiliations:** 1 Rheumatology Division, Central University Hospital of Asturias, 33011 Oviedo, Spain; paulalvarez992@gmail.com (P.A.); stefanie.nam@gmail.com (S.B.); estefaniapardoc@gmail.com (E.P.); i.brana.abascal@hotmail.es (I.B.); mloredomart@gmail.com (M.L.); normaale9222@gmail.com (N.C.); saraalonsocastro@hotmail.com (S.A.); mercedes_alperi@hotmail.com (M.A.); 2 Department of Medicine, Oviedo University School of Medicine, 33006 Oviedo, Spain; 3 Translational Immunology Division, Health Research Institute of the Principality of Asturias (ISPA), 33011 Oviedo, Spain

**Keywords:** psoriatic arthritis, structural damage, disease outcome measures, health impact

## Abstract

**Background/objectives:** Structural joint damage remains a major determinant of long-term disability in psoriatic arthritis (PsA). However, its relationship with current disease activity and patient-reported impact in routine clinical practice is not fully understood. We aimed to assess the prevalence and burden of structural joint damage in PsA and to examine its associations with disease activity, patient-reported impact, and clinical characteristics using complementary analytical approaches. **Methods:** This cross-sectional real-world study included 165 patients with PsA. Structural damage was assessed on conventional radiographs and defined as the presence of at least one joint with erosion, deformity/ankylosis, or joint space narrowing. Damage was analyzed as a binary outcome and as an ordinal burden (0, 1–2, ≥3 affected joints). Disease activity was evaluated using DAPSA, and patient-reported impact using PsAID and the ASAS Health Index (ASAS HI). Multivariable logistics and ordinal regression models were applied. Sensitivity analyses included alternative damage definitions, exclusion of joint space narrowing, restriction to longer disease duration, and adjustment for treatment exposure. **Results:** Structural damage was present in 26.7% of patients. Disease duration was consistently associated with the presence (OR 1.10 per year; 95% CI 1.05–1.15) and increasing burden of structural damage across all analyses. Distal interphalangeal involvement at presentation was strongly associated with higher damage burden (OR 4.29; 95% CI 1.88–9.78). No significant association was observed between structural damage and current disease activity as assessed by DAPSA, while PsAID showed only a non-significant trend. In contrast, ASAS HI scores were significantly higher in patients with structural damage and increased progressively with greater damage burden (ρ = 0.172; *p* = 0.027). Findings remained robust across sensitivity analyses, including restriction to erosive damage and exclusion of joint space narrowing. **Conclusions:** In PsA, structural joint damage is primarily driven by cumulative disease exposure rather than current inflammatory activity. Disease duration and distal interphalangeal involvement identify patients at higher structural risk, while health impact measured by ASAS HI reflects accumulated damage more closely than conventional activity indices.

## 1. Introduction

Psoriatic arthritis (PsA) is a heterogeneous immune-mediated inflammatory disease characterized by variable involvement of the peripheral and axial joints, entheses, digits, skin, and nails. Beyond symptomatic burden, structural joint damage remains a major determinant of long-term disability, impaired function and reduced quality of life [1,2]. Although the treat-to-target paradigm and the broader availability of biologic and targeted synthetic disease-modifying therapies have substantially improved disease control, irreversible damage still occurs in a clinically relevant proportion of patients, and its determinants in routine practice are incompletely understood [1,2].

A key challenge in PsA management is the dissociation between cross-sectional measures of disease activity or patient-reported impact and the accumulated structural burden [3,4,5,6]. Composite activity-indices primarily capture current inflammatory status, whereas radiographic damage reflects the integrated effect of disease duration, past inflammatory burden, mechanical stress and treatment exposure over time [3,4,5,6]. Consequently, patients may exhibit limited clinical activity while already having structural damage or conversely show high symptom impact with little objective damage [7]. This discordance complicates risk stratification and may contribute to missed opportunities for early intervention in patients at higher structural risk.

Several clinical and biological factors have been associated with structural progression in PsA, including longer disease duration, polyarticular phenotype, dactylitis, enthesitis, distal interphalangeal involvement, and markers of more extensive cutaneous disease [3,4,5,6]. Lifestyle and comorbidity factors, particularly smoking, have also been discussed, although their role remains controversial and may be influenced by disease severity and treatment patterns [8]. Importantly, joint space narrowing can partially overlap with degenerative changes, making the interpretation of cartilage loss more complex in real-world cohorts unless supported by standardized scoring and careful phenotyping. In this setting, analyses that separate erosive and deforming damage from joint space narrowing, and that explore structural burden as an ordinal outcome, may provide more robust and clinically meaningful inferences.

While disease duration is a well-established determinant of structural damage in PsA, less attention has been paid to how accumulated damage relates to contemporary measures of disease activity and patient-reported health impact in routine care. In particular, it remains unclear whether commonly used activity indices adequately reflect cumulative structural burden, or whether broader health impact instruments capture dimensions of irreversible joint damage more effectively.

By simultaneously analyzing disease activity (DAPSA), PsA-specific impact (PsAID), and a generic spondyloarthritis health index (ASAS HI) against both the presence and ordinal burden of structural damage, and by testing multiple damage definitions in sensitivity analyses, this study aims to clarify the clinical meaning of these instruments in relation to long-term structural outcomes in PsA.

## 2. Materials and Methods

### 2.1. Study Design and Population

This was a cross-sectional observational study conducted in a real-world cohort of patients with PsA followed at a tertiary rheumatology clinic. A total of 165 consecutive patients with PsA fulfilling the Classification Criteria for Psoriatic Arthritis (CASPAR) and complete clinical, radiographic, and patient-reported data were included in the analysis. All assessments were performed as part of routine clinical care. Clinical, laboratory, radiographic and patient-reported data were extracted from the clinical database and medical records at the time of the study visit. The study was conducted in accordance with the Declaration of Helsinki and was approved by the ethics committee of the Principality of Asturias (Oviedo-Spain) with endorsement #CEImPA 2025.103. Written informed consent was obtained from all participants prior to data collection.

### 2.2. Clinical and Demographic Variables

Demographic variables included age (years), sex, and educational level (low, medium, or higher education). Disease history variables comprised age at onset of psoriasis and arthritis, and disease duration for both conditions, calculated in years. Psoriasis characteristics were recorded using pragmatic clinical proxies, including psoriasis subtype, nail involvement, scalp involvement, intergluteal fold involvement, and extension of cutaneous disease, defined as involvement of more than three body areas. Family history of psoriasis and PsA were recorded as present or absent. Clinical phenotype at presentation and during disease evolution was classified according to standard clinical patterns, including mono-oligoarticular, polyarticular and axial involvement. Specific manifestations such as distal interphalangeal (DIP) joint involvement, dactylitis and enthesitis were recorded as present or absent, both at presentation and during disease evolution. Lifestyle variables included smoking status, which was categorized into never smoker, former smoker and current smoker based on clinical history. Comorbidities were recorded as binary variables and included diabetes mellitus, hypertension, dyslipidemia, overweight, obesity, ischemic heart disease, cerebrovascular disease, and peripheral vascular disease.

### 2.3. Disease Activity and Impact Measures

Tender and swollen joint counts were assessed by the treating rheumatologist during routine visits. Disease activity was quantified using the Disease Activity index for Psoriatic Arthritis (DAPSA) as a continuous variable. Patient-reported impact was assessed using the Psoriatic Arthritis Impact of Disease questionnaire (PsAID) and the ASAS Health Index (ASAS HI), both analyzed as continuous variables. Established thresholds were used descriptively to define low disease activity or low impact, but continuous scores were retained for all statistical analyses. Axial disease activity indices (ASDAS, BASDAI) and PASI were not systematically available for all patients and were therefore not included in the primary analyses.

### 2.4. Treatment Exposure

Current and previous treatments were recorded, including the use of non-steroidal anti-inflammatory drugs, systemic corticosteroids, conventional synthetic disease-modifying antirheumatic drugs (csDMARDs), and biologic disease-modifying antirheumatic drugs (bDMARDs). Exposure to biologic therapies was captured both as a binary variable (ever vs. never) and as the number of biologic therapies used. The number of biologic therapies used was considered a proxy for cumulative disease severity and treatment exposure rather than a direct measure of treatment timing.

### 2.5. Structural Damage Assessment

All radiographic findings were interpreted in patients with confirmed PsA, and structural damage was attributed to PsA based on the overall clinical phenotype and radiographic patterns evaluated during routine rheumatology care. Peripheral joints assessed included hands and feet using conventional radiographs obtained as part of routine clinical care. A formal standardized scoring system such as the PsA-modified Sharp/van der Heijde score was not applied, and no formal blinding procedures were implemented, reflecting the pragmatic real-world design of the study. For each patient, the number of joints showing erosion, deformity/ankylosis, and joint space narrowing (JSN), was recorded by experienced rheumatologists using predefined operational criteria as part of routine clinical practice. Structural damage was analyzed using several complementary definitions: (i) global structural damage (binary outcome), defined as the presence of at least one joint with erosions, deformity/ankylosis or JSN; (ii) damage subtypes (binary outcomes) such as erosive damage, deformity/ankylosis, and JSN were also analyzed separately; and (iii) structural damage burden (ordinal outcome), defined as the total number of affected joints (sum of erosions, deformity/ankylosis and, when applicable, JSN), and categorized into: 0 affected joints, 1–2 affected joints, and ≥3 affected joints. To minimize potential overlap with degenerative changes, additional analyses were performed excluding JSN and restricting the outcome to erosive damage only.

### 2.6. Statistical Analysis

Continuous variables are presented as medians with interquartile ranges (IQR), and categorical variables as absolute frequencies and percentages. Comparisons between patients with and without structural damage were performed using the Mann–Whitney U test for continuous variables and the χ^2^ test or Fisher’s exact test for categorical variables, as appropriate. Associations between structural damage burden and disease activity or patient-reported impact measures (DAPSA, PsAID and ASAS HI) were explored using Spearman’s rank correlation coefficients. Multivariable analyses were conducted using binary logistic regression for the presence of global structural damage, and ordinal logistic regression for increasing categories of structural damage burden. Covariates were selected a priori based on clinical relevance and included age, sex, disease duration, clinical phenotype, psoriasis extension, smoking status and treatment exposure. Additional multivariable models were fitted including DAPSA as a continuous covariate to evaluate whether current disease activity was independently associated with structural damage after adjustment. Given the unequal distribution of patients with and without structural damage, multivariable logistic and ordinal regression models were prioritized to account for potential imbalance between groups. In addition, multiple sensitivity analyses were conducted to assess the robustness of the findings across alternative damage definitions and analytical scenarios. Results are presented as odds ratios (OR) with 95% confidence intervals (CI). A series of sensitivity analyses were pre-specified and performed to assess the robustness of the findings. These included alternative definitions of structural damage (erosions only; exclusion of JSN), restriction to patients with longer disease duration, and models accounting for or excluding treatment exposure. All analyses were performed using standard statistical software, and two-sided *p* values < 0.05 were considered statistically significant.

## 3. Results

### 3.1. Patient Characteristics

A total of 165 patients with PsA were included in the analysis. Overall, 44 patients (26.7%) presented evidence of structural joint damage as previously defined. The remaining 121 patients (73.3%) had no radiographic structural damage. Patients with structural damage had a significantly longer disease duration compared with those without damage (median 12.0 [IQR 5.5–22.5] vs. 6.0 [2.0–12.0] years; *p* < 0.001). Age showed a non-significant trend towards higher values in patients with damage (*p* = 0.075), while sex distribution was similar between groups (*p* = 1.00). No significant differences were observed between patients with and without structural damage regarding psoriasis extension (>3 body areas), nail involvement, scalp involvement, or intergluteal fold involvement. Similarly, no significant differences were detected for polyarticular phenotype, history of dactylitis or enthesitis, or smoking status when analyzed as categorical variables. Detailed baseline characteristics are shown in Table 1.

### 3.2. Prevalence and Patterns of Structural Damage

Structural joint damage was present in 44 patients (26.7%). The prevalence of overall damage, individual damage subtypes, and the distribution of structural damage burden are summarized in Table 2.

### 3.3. Structural Damage and Current Disease Activity and Patient-Reported Impact

Current peripheral disease activity, assessed by DAPSA, did not differ significantly between patients with and without structural damage (median 8.2 [3.3–15.0] vs. 9.4 [6.1–14.2]; *p* = 0.46). Similarly, PsAID scores showed no statistically significant difference between groups (2.5 [0.85–4.95] vs. 3.85 [1.64–5.05]; *p* = 0.11). In contrast, patients with structural damage reported significantly worse health status as measured by the ASAS HI (6.0 [5.0–10.0] vs. 4.0 [2.0–8.0]; *p* = 0.017). The number of swollen joints was also slightly higher in patients with damage (*p* = 0.012), although absolute differences were small.

**Figure 1:**
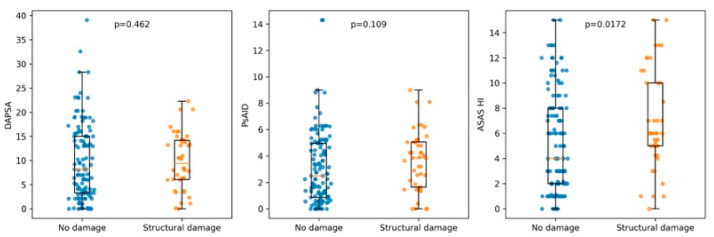
Distribution of disease activity and patient-reported impact measures according to the presence of structural damage. Boxplots show median and interquartile range, with individual patient values overlaid. Structural damage was defined as the presence of at least one joint with erosions, deformity/ankylosis, or joint space narrowing. Comparisons between groups were performed using the Mann–Whitney U test. DAPSA and PsAID showed no significant differences between patients with and without structural damage, whereas ASAS Health Index scores were significantly higher in patients with structural damage.

When analyzing damage as a continuous burden (number of affected joints), no significant correlation was observed with DAPSA (ρ = 0.036; *p* = 0.64) or PsAID (ρ = 0.108; *p* = 0.17). In contrast, a weak but statistically significant correlation was found between damage burden and ASAS HI (ρ = 0.166; *p* = 0.033), as well as with the number of swollen joints (ρ = 0.201; *p* < 0.01). These relationships are illustrated in Figure 1.

### 3.4. Factors Associated with the Presence of Structural Damage

**Figure 2:**
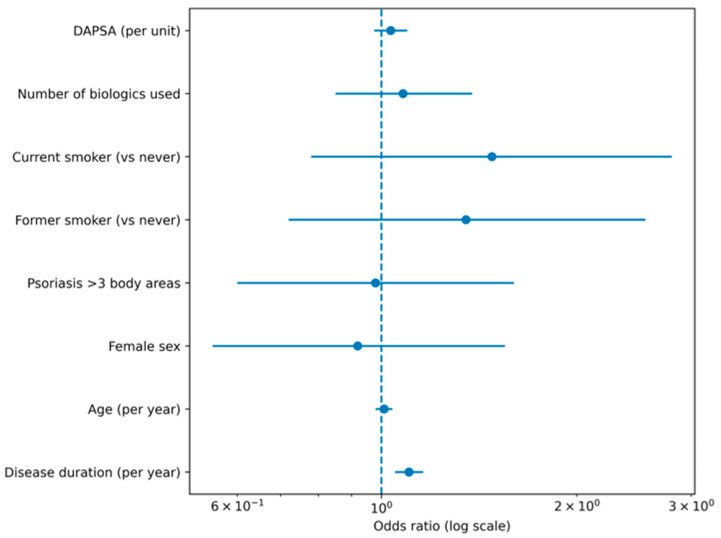
Multivariable logistic regression model of factors associated with structural damage. Forest plot showing odds ratios (OR) and 95% confidence intervals from a multivariable logistic regression model evaluating factors associated with the presence of structural damage. Structural damage was defined as the presence of at least one joint with erosions, deformity/ankylosis, or joint space narrowing. The vertical dashed line indicates the null effect (OR = 1).

In multivariable logistic regression analysis, structural damage (binary outcome) was independently associated with longer disease duration. Each additional year of arthritis duration was associated with a 10% increase in the odds of structural damage (OR 1.10, 95% CI 1.05–1.15; *p* < 0.001). Age, sex, polyarticular phenotype, psoriasis extension (>3 body areas), smoking status (former or current vs. never), and number of biologic therapies used were not independently associated with the presence of structural damage in the adjusted model. When DAPSA was included as a covariate in the multivariable logistic regression model, it was not independently associated with structural damage (OR 1.03 per unit increase; 95% CI 0.97–1.10; *p* = 0.268). Disease duration remained robustly associated with structural damage (OR 1.10 per year; 95% CI 1.05–1.16; *p* < 0.001). The results of the multivariable logistic regression analysis are summarized in Figure 2.

### 3.5. Structural Damage Burden: Ordinal Analysis

**Figure 3:**
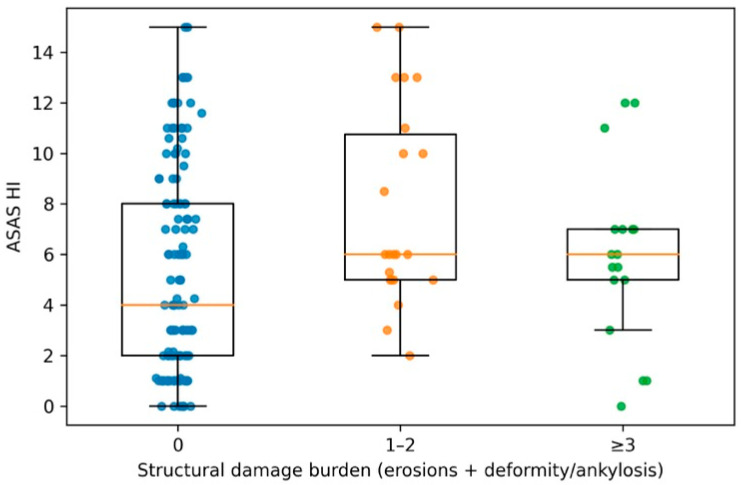
Distribution of ASAS HI scores according to structural damage burden. Distribution of ASAS Health Index scores according to increasing categories of structural damage burden. Structural damage burden was defined as the number of joints with erosions or deformity/ankylosis and categorized as 0, 1–2, or ≥3 affected joints. Boxplots show median and interquartile range, with individual patient values overlaid. Joint space narrowing was excluded from this analysis.

To further explore the relationship between cumulative damage and clinical characteristics, structural damage burden was analyzed as an ordinal outcome (0, 1–2, ≥3 affected joints). A clear dose–response relationship was observed between increasing damage burden and disease duration (ρ = 0.307; *p* < 0.001). Patients with higher damage categories also showed progressively worse ASAS HI scores (ρ = 0.172; *p* = 0.027) and a higher number of swollen joints (ρ = 0.204; *p* < 0.01). No significant trend was observed for DAPSA or PsAID across damage categories. Distal interphalangeal (DIP) joint involvement at presentation showed a strong association with increasing damage burden, with proportions rising from 22.3% in patients without damage to 70.8% in those with ≥3 damaged joints (*p* for trend <0.001). In ordinal multivariable regression analysis, both disease duration (OR 1.10 per year; 95% CI 1.05–1.15; *p* < 0.001) and DIP involvement at presentation (OR 4.29; 95% CI 1.88–9.78; *p* < 0.001) remained independently associated with higher damage burden, whereas age, sex, smoking status and biologic treatment exposure were not. These findings are summarized in Figure 3.

### 3.6. Sensitivity Analyses

When structural damage was restricted to erosive lesions only, disease duration remained significantly associated with damage (OR 1.07 per year; *p* < 0.01), and distal interphalangeal involvement at presentation showed a strong association with erosive damage (OR 4.62; *p* < 0.001). Excluding joint space narrowing from the damage definition did not materially alter the results, with disease duration (OR 1.10 per year; *p* < 0.001) and distal interphalangeal involvement (OR 3.89; *p* < 0.01) remaining significantly associated with structural damage. When the analysis was restricted to patients with a disease duration of at least 2 years (n = 141), disease duration remained independently associated with structural damage (OR 1.10 per year; *p* < 0.001), and distal interphalangeal involvement at presentation continued to show a significant association (OR 2.87; *p* = 0.017) while psoriasis extension (>3 body areas) was not associated with damage. Finally, analyses restricted to patients treated with ≤1 biologic therapy yielded consistent results, with disease duration (OR 1.09 per year; *p* < 0.01) and DIP involvement (OR 8.41; *p* < 0.001) remaining strongly associated with structural damage.

## 4. Discussion

The main contribution of this study lies not in identifying novel predictors of structural damage, but in clarifying how different dimensions of disease assessment—current inflammatory activity, patient-reported impact, and cumulative structural burden—relate to each other in real-world PsA. By combining binary and ordinal damage outcomes with complementary activity and impact measures, our findings highlight the limitations of cross-sectional activity indices and underscore the added value of broader health status instruments, particularly the ASAS Health Index, in reflecting accumulated structural involvement.

The prevalence of structural damage observed in our cohort is broadly consistent with previous real-world studies, supporting the notion that irreversible joint damage remains a clinically relevant issue in PsA even in the biologic era [3,4,5,6]. Moreover, this damage was not as extensive and severe as that recorded in historical PsA series [9,10,11], which may reflect the improvements in diagnosis and treatment introduced in recent years. In our series, damage was not restricted to isolated lesions but showed meaningful burden in a subset of patients, with more than one in ten patients presenting three or more structurally damaged joints. This finding underscores that structural involvement is not merely a historical artifact of untreated disease, but rather a persisting outcome in routine clinical practice, despite the wide range of current treatments for the disease. By analyzing damage subtypes separately, we confirmed that erosive and deforming/ankylosing changes constitute a substantial proportion of the overall structural burden, while JSN accounts for a smaller but non-negligible component. The consistency of our findings after excluding JSN reinforces that the observed associations are not driven by potential overlaps with degenerative changes, and strengthens the inflammatory specificity of our results.

Across all analytical strategies, disease duration emerged as the most consistent and robust factor associated with both the presence and increasing burden of structural damage. Each additional year of arthritis duration conferred a measurable increase in the odds of damage, and this association was further strengthened when analyses were restricted to patients with longer-standing disease. These findings align with the concept that structural damage in PsA reflects cumulative inflammatory burden over time rather than short-term disease fluctuations [12]. Notably, age per se did not retain an independent association with damage after adjustment, suggesting that chronological aging is less relevant than the duration of inflammatory exposure. This distinction is clinically important, as it emphasizes the need for early disease recognition and timely therapeutic intervention to mitigate long-term structural consequences.

Distal interphalangeal (DIP) joint involvement at presentation showed a particularly strong association with increasing structural damage burden, including in analyses restricted to erosive damage. This observation supports the notion that DIP involvement may represent a marker of a more aggressive structural phenotype in PsA [13,14]. The association persisted across multiple models and sensitivity analyses, highlighting its potential relevance for risk stratification. The strong association between DIP involvement and structural damage is biologically plausible. The DIP joint represents a unique anatomical unit integrating the nail apparatus, entheses, and adjacent bone, which may predispose to combined erosive and proliferative changes. Biomechanical stress and synovio-entheseal complex involvement have been proposed as key mechanisms linking DIP disease to structural damage in PsA [14,15]. While causality cannot be inferred from our cross-sectional design, the consistency of this association suggests that the early identification of DIP involvement may help identify patients at higher risk of cumulative structural damage.

A central finding of our study is the weak or absent association between structural damage and current disease activity as assessed by DAPSA. Neither the presence nor the burden of damage correlated meaningfully with contemporaneous disease activity scores, reinforcing the concept that activity indices primarily capture current inflammatory status rather than accumulated structural burden [7,12]. This dissociation has important clinical implications. Patients with well-controlled disease activity may already harbor significant structural damage, while others with active symptoms may have little irreversible joint involvement. These findings caution against relying exclusively on cross-sectional activity measures to infer long-term structural risk and support the need for complementary assessment strategies.

In contrast to activity indices, patient-reported health impact measured by the ASAS HI showed a modest but consistent association with both the presence and increasing burden of structural damage. Although the magnitude of this association was limited, it remained statistically significant and displayed a clear dose–response pattern in ordinal analyses. The ASAS HI captures a broader construct of functioning and health status, integrating physical limitations, pain, fatigue and psychosocial aspects [16]. Its association with structural damage likely reflects the cumulative functional consequences of irreversible joint changes rather than acute inflammatory activity. Importantly, this relationship was not observed for PsAID to the same extent. This discrepancy may reflect domain-level differences between instruments. ASAS HI captures functional limitations and broader aspects of health status that are more closely related to irreversible structural damage, whereas PsAID places greater emphasis on symptom burden and skin-related impact, which may fluctuate independently of cumulative joint damage. These findings support the complementary value of ASAS HI in PsA, particularly when structural outcomes are of interest, and suggest that it may serve as a useful proxy for accumulated disease burden in cross-sectional settings.

From a treat-to-target perspective, these findings suggest that disease activity indices such as DAPSA may not fully capture long-term structural risk. Periodic structural assessment and complementary health impact measures such as ASAS HI may therefore add value in selected patients, particularly those with long-standing disease or features associated with higher structural burden.

Smoking exposure showed a graded association with increasing damage burden in unadjusted and ordinal analyses, although this relationship did not consistently retain statistical significance in fully adjusted models. This attenuation likely reflects limited power and residual confounding, rather than the absence of a true biological effect. Smoking has been implicated in inflammatory arthritis severity and treatment response, and its potential contribution to structural damage in PsA warrants further investigation in longitudinal studies [17,18,19]. Nevertheless, the observed trends in our data reinforce the relevance of smoking as a modifiable risk factor in the comprehensive management of PsA.

The number of biologic therapies used did not emerge as an independent determinant of structural damage in adjusted analyses. Importantly, sensitivity analyses excluding treatment exposure or restricting the cohort to patients with limited biologic use yielded consistent results. These findings suggest that the observed associations are not primarily driven by confounding by indication. It should be emphasized that treatment exposure in this context is likely to reflect disease severity and therapeutic history rather than a causal effect on damage development. Our results therefore support the interpretation of biologic use as a marker of cumulative disease burden rather than a determinant of structural outcomes in cross-sectional analyses.

Several methodological strengths enhance the robustness of our findings. We employed multiple complementary definitions of structural damage, including binary and ordinal outcomes, and conducted extensive sensitivity analyses to address potential sources of bias, particularly the contribution of joint space narrowing. On the other hand, the unequal size of the damaged and non-damaged groups reflects the true prevalence of structural involvement in contemporary real-world PsA cohorts rather than a selection bias. Importantly, the consistency of results across adjusted and sensitivity analyses supports the robustness of our findings despite this imbalance.

Several limitations should be acknowledged. The cross-sectional design precludes causal inference, and associations observed—particularly regarding DIP involvement—should not be interpreted as predictive. Structural damage was assessed using routine radiographs, which may under detect early or subtle changes compared with more sensitive imaging modalities such as ultrasound or MRI. Although axial activity indices such as BASDAI or ASDAS were not systematically available and could not be included, the consistency of our findings across peripheral activity measures and multiple sensitivity analyses suggests that the observed dissociation between current activity and cumulative damage is unlikely to be fully explained by the absence of axial indices. Both joint counts and radiographic damage assessment are subject to inter-observer variability, as they rely on clinical judgement in routine practice. Although formal inter-reader reliability testing was not performed, this pragmatic approach reflects real-world care and may bias associations toward the null rather than generating spurious positive findings. Additionally, although our cohort size is comparable to many real-world PsA studies, limited sample size may have reduced the power to detect weaker associations, particularly for lifestyle and comorbidity factors. Residual confounding by unmeasured variables, including historical inflammatory burden and treatment timing, cannot be excluded. Finally, detailed data on treatment timing, such as time since biologic initiation or duration of uncontrolled disease prior to treatment, were not available and could not be accounted for in the analysis.

Our findings have several practical implications for PsA management. First, they reinforce the importance of early diagnosis and sustained disease control to limit cumulative inflammatory exposure and subsequent structural damage. Second, they highlight the limitations of relying solely on cross-sectional activity measures to assess long-term structural risk. Third, they support the use of complementary patient-reported outcomes, such as ASAS HI, to capture aspects of disease impact related to irreversible damage. From a clinical perspective, these results argue for additional assessment strategies including periodic structural imaging, integration of functional outcome measures, and the use of broader health impact instruments to complement activity-based indices, particularly in patients with long-standing disease. In addition, these findings should be interpreted within the context of current management principles in PsA, which emphasize early diagnosis, sustained disease control, and periodic assessment of structural damage. Our results reinforce these principles by highlighting the dissociation between current activity and cumulative structural burden, rather than proposing new management targets.

## 5. Conclusions

Despite the current therapeutic armamentarium, structural joint damage remains prevalent in PsA and is primarily associated with cumulative disease exposure rather than current inflammatory activity. Disease duration and distal interphalangeal involvement emerged as key markers of structural burden, while patient-reported health impact captured by ASAS HI reflected accumulated damage more closely than conventional activity indices and standard impact measures such as PsAID. These findings underscore the need for long-term perspectives in PsA management and support the integration of structural and functional assessments in routine care.

## Figures and Tables

**Table 1:** Baseline characteristics according to presence of structural damage.

Characteristic	Total Cohort (n = 165)	No Structural Damage (n = 121)	Structural Damage (n = 44)	*p* Value
Demographics				
Age, years, median (IQR)	55 (45–64)	54 (44–63)	58 (48–67)	0.075
Female sex, n (%)	87 (52.7)	64 (52.9)	23 (52.3)	1.00
Disease history				
Age at arthritis onset, years, median (IQR)	45 (36–55)	46 (37–55)	43 (33–54)	0.41
Disease duration, years, median (IQR)	7.0 (3.0–14.0)	6.0 (2.0–12.0)	12.0 (5.5–22.5)	<0.001
Psoriasis characteristics				
Psoriasis > 3 body areas, n (%)	63 (38.2)	46 (38.0)	17 (38.6)	0.83
Nail involvement, n (%)	72 (43.6)	52 (43.0)	20 (45.5)	0.86
Scalp involvement, n (%)	83 (50.3)	60 (49.6)	23 (52.3)	0.77
Intergluteal fold involvement, n (%)	39 (23.6)	28 (23.1)	11 (25.0)	0.83
Clinical phenotype				
Polyarticular pattern (ever), n (%)	69 (41.8)	49 (40.5)	20 (45.5)	0.59
DIP involvement at presentation, n (%)	56 (33.9)	27 (22.3)	29 (65.9)	<0.001
History of dactylitis, n (%)	47 (28.5)	31 (25.6)	16 (36.4)	0.18
History of enthesitis, n (%)	52 (31.5)	36 (29.8)	16 (36.4)	0.43
Lifestyle				
Never smoker, n (%)	78 (47.3)	71 (58.7)	7 (15.9)	-
Former smoker, n (%)	46 (27.9)	29 (24.0)	17 (38.6)	-
Current smoker, n (%)	41 (24.8)	21 (17.3)	20 (45.5)	0.185 ^†^
Treatment exposure				
Current biological therapy, n (%)	92 (55.8)	66 (54.5)	26 (59.1)	0.61
Number of biologic therapies used, median (IQR)	1 (0–2)	1 (0–2)	1 (1–2)	0.27
Disease activity and impact				
Tender joint count, median (IQR)	1 (0–4)	1 (0–4)	2 (0–5)	0.21
Swollen joint count, median (IQR)	0 (0–0)	0 (0–0)	0 (0–1)	0.012
DAPSA, median (IQR)	8.6 (3.6–14.9)	8.2 (3.3–15.0)	9.4 (6.1–14.2)	0.46
PsAID, median (IQR)	2.9 (1.0–5.0)	2.5 (0.9–4.9)	3.9 (1.6–5.1)	0.11
ASAS HI, median (IQR)	5.0 (2.0–9.0)	4.0 (2.0–8.0)	6.0 (5.0–10.0)	0.017

^†^ *p* value corresponds to comparison of smoking status as a categorical variable (never/former/current). Values are presented as median (interquartile range) or number (%), as appropriate. Structural damage was defined as the presence of at least one joint with erosion, deformity/ankylosis or joint space narrowing. Comparisons were performed using the Mann–Whitney U test for continuous variables and the χ^2^ test or Fisher’s exact test for categorical variables.

**Table 2:** Prevalence and patterns of structural joint damage in the study cohort (n = 165).

Structural Damage Characteristic	n (%)
Any structural damage	44 (26.7)
Damage subtypes	
Erosive damage	32 (19.4)
Deformity/ankylosis	27 (16.4)
Joint space narrowing (JSN)	24 (14.5)
Structural damage burden	
0 affected joints	121 (73.3)
1–2 affected joints	20 (12.1)
≥3 affected joints	24 (14.5)

Structural damage was defined as the presence of at least one joint with erosion, deformity/ankylosis, or joint space narrowing (JSN) on conventional radiographs. Damage subtypes were not mutually exclusive. Structural damage burden was defined as the total number of affected joints and categorized as 0, 1–2, or ≥3 joints.

## Data Availability

The data presented in this study are available on request from the corresponding author. Due to patient confidentiality and institutional policies, raw individual-level data cannot be made publicly available.
